# Determinants of Health‐Seeking Behavior and Quality of Life in Patients With Noncommunicable Diseases in Bangladesh

**DOI:** 10.1002/puh2.70238

**Published:** 2026-04-21

**Authors:** Kazi Abdus Sobur, MD. Faisal Ahmed, Gazi Mahjabin Islam, Sanjana Islam, Saiyada Hossain Saima

**Affiliations:** ^1^ Department of Health Sciences and Informatics Bangladesh Institute of Innovative Health Research, Mirpur Dhaka Bangladesh

**Keywords:** healthcare disparities, health‐seeking behavior, noncommunicable diseases, preventive care, quality of life

## Abstract

**Background:**

Noncommunicable diseases (NCDs) are the leading cause of mortality in Bangladesh, yet healthcare access and quality of life (QoL) remain suboptimal. Unlike previous studies in Bangladesh, this research systematically applies Andersen's model to jointly assess health‐seeking behavior (HSB) and QoL, offering a novel framework for policy relevant insights.

**Methods:**

A cross‐sectional survey was conducted from November 2024 to February 2025 among 1052 adults with clinically confirmed NCDs in urban and rural areas of Dhaka, Khulna, and Mymensingh. Data on sociodemographic, HSB, and QoL (Bangla WHOQoL‐BREF) were collected. Chi‐square tests, ANOVA, and multiple regression identified predictors of HSB and QoL.

**Results:**

Among 1052 participants, hypertension (36%), diabetes (24%), and cardiovascular disease (18%) were most common. Over half (58%) sought care only in emergencies, mainly due to financial (48%) and access (21%) barriers. Education strongly predicted proactive HSB (*p* < 0.001), whereas employment was negatively associated with follow‐up and visits (*p* < 0.01). Socioeconomic status predicted higher QoL (*β* = 0.174 and *p* < 0.001), and rural residents reported significantly lower QoL than urban counterparts (*p* < 0.001). Men had better psychological health than women (*p* < 0.001). Overall, findings highlight socioeconomic, occupational, and geographic disparities in care‐seeking and QoL.

**Conclusion:**

Patients with NCDs in Bangladesh face delayed care‐seeking, rural–urban disparities, and low uptake of lifestyle modifications. Interventions should prioritize community‐based screening, financial protection schemes, and health literacy programs to improve preventive care and QoL.

## Graphical abstract

Determinants of health‐seeking behavior and quality of life in patients with NCDs in Bangladesh.

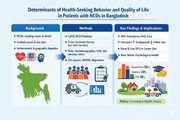

## 1 Introduction

Noncommunicable diseases (NCDs) have emerged as a leading cause of death globally and are accountable for 75% of all deaths, thus, contributing to approximately 43 million deaths per year [1, 2]. These diseases, such as cardiovascular diseases, cancers, chronic respiratory diseases, and diabetes, are generally defined by long‐term, progressive, and noncommunicable conditions resulting from multiple gene‐environment and behavior interactions [3]. It was estimated that high‐income countries had a greater NCD toll in the past; however, low‐ and middle‐income countries (LMICs) such as Bangladesh saw a steep progression in the incidence and death rates of NCD in the last few decades [4]. Currently, NCDs have become the cause of more than 70% of mortality in Bangladesh; hence, the emphasis is now on specific NCD management and prevention techniques [5].

There are system attributes that have fostered the rise of NCDs in LMICs, such as a lack of/enabling healthcare facilities, poor/enabling healthcare financing, and disability‐adjusted life years due to socioeconomic status. Moreover, other modifiable risk factors, including active smoking, inactivity, increased salt‐saturated diet, and excessive alcohol intake, contribute to NCD risk and disease progression [6]. Bangladesh's health system still prioritizes infectious diseases, leaving chronic NCD care under‐resourced. A transition from contagious diseases to longevity diseases as the leading cause of death has left the healthcare system ill‐equipped to deal with NCDs [7]. The NCD situation in Bangladesh reveals some challenges that complicate its management and prevention. Health‐seeking behavior (HSB) is a key determinant of NCD management, defined as the efforts that people make in an attempt to gain proactive and curative access to healthcare for themselves [8]. HSB represents a spectrum of patients’ interactions with healthcare, ranging from symptom awareness to diagnosis search and treatment compliance. Hypertension, diabetes, stroke, and other associated cardiovascular and chronic respiratory diseases require frequent follow‐up, strict adherence to medications, and alteration of lifestyles for the best results [3, 9].

However, some factors hinder HSB among patients with NCD and limit them from seeking care or receiving the right treatment in Bangladesh. The inability to get access to healthcare facilities owing to poverty or inadequate insurance is another explanation, added to the inability to access indispensable medications [10, 11]. Other barriers include cultural beliefs, social stigma, and low health literacy, which predispose the affected persons to the symptoms, but they are unable to seek early diagnosis or intervention [12]. For example, culture and tradition, such as gender roles, restrain women's movement and HSB without a chaperone, which delays needed care [13]. Further, the rural population has even higher barriers to access health facilities than their urban counterparts, as most of the facilities available are located in urban centers [14]. Using patients’ own words, self‐reported quality of life (QoL) in patients with NCD is intimately associated with their capacity to be active in managing their illness. In many patients, NCDs’ chronic nature and the associated psychosocial impact drastically reduce their QoL. Healthcare accessibility hitches, along with poor symptom control, pose potential threats to QoL as they enhance morbidity or risk of disease complications [15]. Despite the growing literature, little is known about how socioeconomic and cultural determinants interact to shape both HSB and QoL within the same analytical framework in Bangladesh. Addressing this gap is crucial for context‐specific interventions. Therefore, it is essential to identify the determinants and correlates of HSB and QoL of the patients with NCD in Bangladesh to enhance the general patient profile endowed with efficient growth of health strategies to aid the illnesses.

Recent studies indicate an emerging trend of early‐age NCD diagnosis in South Asia, partly attributed to rapid urbanization, sedentary lifestyles, and dietary transitions [16, 17, 18, 19]. However, epidemiological data still suggest that older adults bear the highest NCD burden, raising concerns when facility‐based studies report unusually high proportions of young patients—such as in the present study, where over 40% of participants were aged 18–24 years. This pattern requires careful interpretation as it may reflect urban facility concentration, student population bias, or evolving epidemiological trends. Research focusing on health behaviors and QoL of patients with NCD in Bangladesh is valuable because the country has one of the highest mortality rates due to NCDs in South Asia; moreover, massive migrations from rural areas to urban ones or other countries have created severities in terms of health facility access due to the nature of employment opportunities and cultural specificity of the Bangladeshi population [20]. Other prevalence studies have focused on brief emergent health system issues and NCD but have not adequately explored the factors that affect HSB and QoL in Bangladeshi patients with NCD [21]. To address this gap, the present study is informed by Andersen's Behavioral Model of Health Services Use, which theorizes that HSB is shaped by predisposing characteristics (e.g., age and gender), enabling resources (e.g., income and access), and need factors (e.g., illness severity) [22]. Although international strategies guide the management of NCDs, they are often not directly applicable to the sociocultural and economic realities of countries like Bangladesh [23].

Andersen's Behavioral Model provides a theoretically robust framework for jointly analyzing HSB and QoL because it integrates structural determinants, enabling resources, and perceived need factors within a unified explanatory system. These domains simultaneously influence whether individuals seek care and how health conditions affect their lived well‐being. Prior studies in Bangladesh and similar LMIC contexts have largely examined NCD prevalence or healthcare utilization independently, without applying a comprehensive behavioral model to explain both utilization patterns and outcome experiences. By embedding both HSB and QoL within Andersen's framework, the present study enables a more holistic understanding of how socioeconomic, demographic, and system‐level determinants interact to shape patient behavior and health outcomes.

## 2 Materials and Methods

### 2.1 Ethical Consideration

Ethical approval was obtained from the Institutional Review Board (IRB) at the Bangladesh Institute of Innovative Health Research before data collection (IRB Protocol No.: BIIHR‐2024‐017). The study adhered to the guidelines provided in the Declaration of Helsinki [24].

### 2.2 Study Design and Settings

A cross‐sectional survey was conducted between November 2024 and February 2025 across three administrative divisions—Dhaka, Khulna, and Mymensingh. Both urban wards (e.g., Dhaka City Corporation) and rural unions (e.g., Savar, Dumuria, and Trishal) were purposively selected to capture variation in socioeconomic and healthcare access contexts.

### 2.3 Study Participants

Eligible participants were adults (≥18 years) with a clinically confirmed diagnosis of at least one noncommunicable disease (hypertension, diabetes, cardiovascular disease, chronic respiratory disease, or cancer) and a treatment history of ≥6 months. Exclusion criteria included acute illness and inability to provide informed consent. Diagnoses were verified through medical records, prescriptions, or ongoing medication use. Using stratified purposive sampling by division (urban/rural), 1052 participants were enrolled. The required sample size was estimated at 385 using Cochran's formula; the achieved sample provided adequate statistical power.

### 2.4 Measures

Data were collected using a structured, interviewer‐administered questionnaire with four sections:

Sociodemographics: age, gender, education, occupation, marital status, income, and residence.

NCD profile: diagnosis, duration, treatment type, and comorbidities.

**FIGURE 1:**
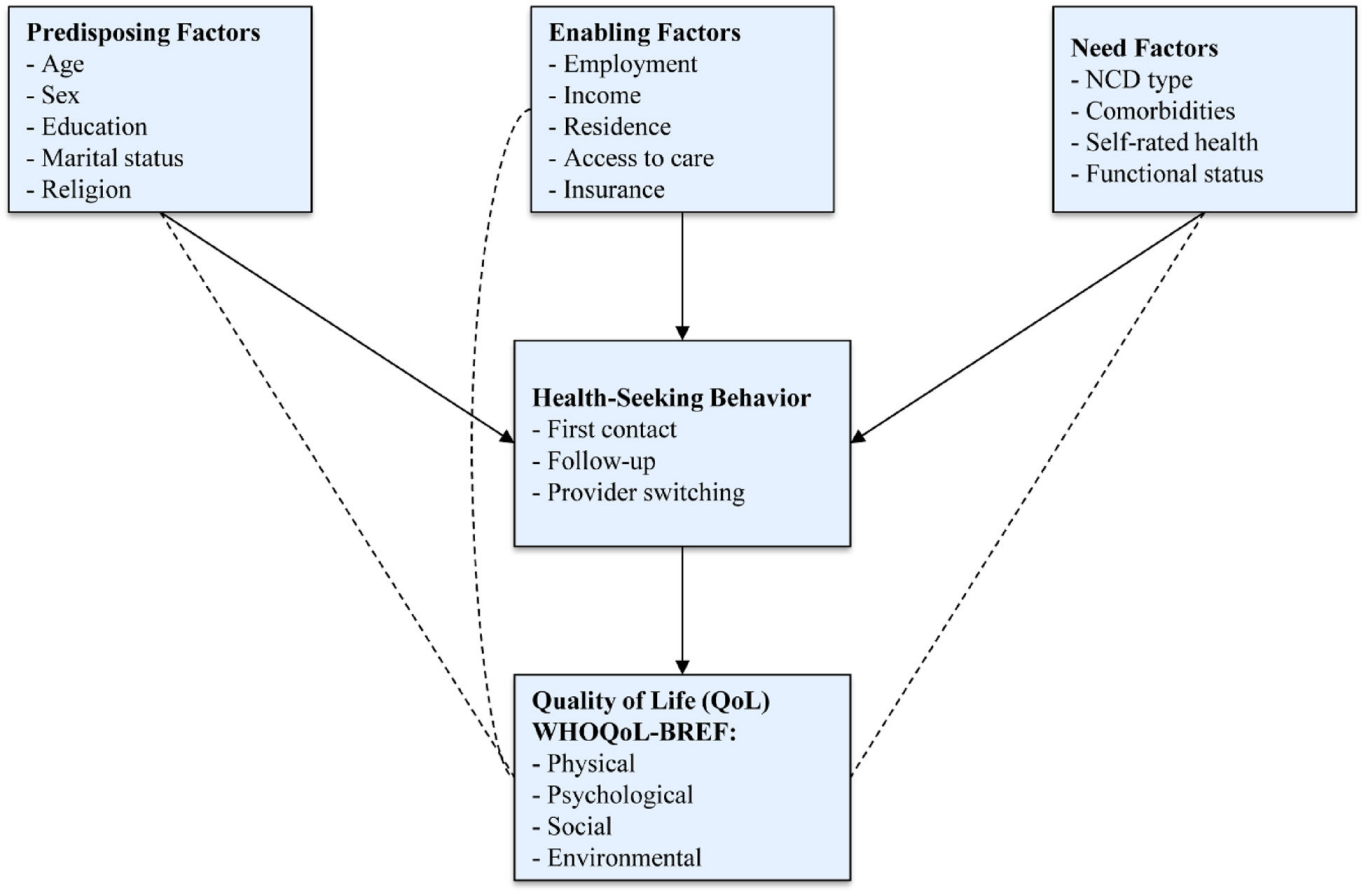
Conceptual framework based on Andersen's Behavioral Model of Health Services Use. *Source:* Adapted from Andersen [22]. NCD, noncommunicable disease.

HSB: frequency of visits, provider type, barriers to access, and reasons for delaying care, adapted from Andersen's Behavioral Model (Figure 1) [22].

QoL: assessed using the validated Bangla WHOQoL‐BREF [25, 26], covering physical, psychological, social, and environmental domains. In this study, Cronbach's alpha ranged from 0.78 to 0.86 across domains.

The full questionnaire, including translated items, is available in Supporting Information File 1.

STROBE checklist for cross‐sectional studies is available in Supporting Information File 2.

### 2.5 Data Collection

Trained research assistants conducted face‐to‐face interviews at community sites. Interviews lasted 20–30 min and were pilot‐tested with 30 participants for clarity and cultural appropriateness. Interviewer administration was chosen to minimize literacy‐related bias and ensure standardized data collection.

### 2.6 Statistical Analysis

Data were analyzed in SPSS v26. Descriptive statistics summarized participant characteristics. Chi‐square tests assessed associations between categorical variables; independent *t*‐tests and ANOVA compared mean scores across groups. Variables with *p* < 0.05 in bivariate analysis were included in multivariate regression models for HSB and QoL outcomes. Categorical predictors were dummy‐coded, with the most common group as reference. Multicollinearity was assessed using VIF (<5 acceptable). Model fit was evaluated using adjusted *R* ^2^, Akaike information criterion (AIC), and Bayesian information criterion (BIC). Missing data (<5%) were handled with listwise deletion. Statistical significance was set at *p* < 0.05. Detailed regression diagnostics are presented in Tables S1 and S2.

## 3 Results

This study analyzed data to explore the factors influencing HSB and QoL among patients with NCDs in Bangladesh.

### 3.1 Sociodemographic Characteristics

The sample was nearly gender‐balanced (51% male and 49% female). More than three‐quarters (77.0%) resided in urban areas, and 80.8% reported belonging to the middle socioeconomic class. A total of 600 participants (57.0%) had completed graduate‐level education or higher. The largest age group comprised individuals aged 18–24 years (42.5%), whereas only 5.1% were aged 65 years or older (Table 1). This unusually young age profile may reflect sampling from urban and peri‐urban populations, where health awareness and healthcare utilization tend to be higher among younger adults.

**TABLE 1:** Sociodemographic characteristics of study participants (*n* = 1052).

Category	Variable	Frequency	Percentage
**Age (years)**	18–24	447	42.5
	25–34	262	24.9
	35–44	132	12.5
	45–54	92	8.7
	55–64	65	6.2
	65 and older	54	5.1
**Gender**	Male	535	50.9
	Female	517	49.1
**Educational background**	No formal education	70	6.7
	Primary	47	4.5
	Secondary	69	6.6
	Higher secondary	266	25.3
	Graduate and above	600	57.0
**Socioeconomic status**	Lower class	119	11.3
	Middle class	850	80.8
	Higher class	83	7.9
**Occupation**	Unemployed	60	5.7
	Student	472	44.9
	Homemaker	189	18.0
	Employed	285	27.1
	Retired	46	4.4
**Marital status**	Single	543	51.6
	Married	477	45.3
	Divorced	17	1.6
	Widowed	15	1.4
**Residential area**	Urban	810	77.0
	Rural	242	23.0

### 3.2 Clinical Characteristics

The most frequently reported NCD was hypertension (36.2%), followed by diabetes (24.1%) and cardiovascular diseases (17.6%) (Table 2). Approximately 50.5% of participants had been diagnosed within the past 2 years, whereas 56.4% reported using medication as the primary treatment modality. Notably, 54.3% sought medical follow‐ups only when they perceived a need rather than on a regular basis, indicating a potential gap in preventive care utilization.

**TABLE 2:** Clinical characteristics of study participants (*n* = 1052).

Category	Variable	Frequency	Percentage
**Primary NCD diagnosis**	Diabetes	254	24.1
	Hypertension	381	36.2
	Cardiovascular disease	185	17.6
	Cancer	17	1.6
	Chronic respiratory disease	53	5.0
	Others	162	15.4
**Duration of illness (years)**	0–2	531	50.5
	3–5	331	31.5
	6–8	94	8.9
	9 or more	96	9.1
**Current treatment type**	Medication	593	56.4
	Lifestyle modification	305	29.0
	Surgery	34	3.2
	Others	120	11.4
**Frequency of medical follow‐ups**	Monthly	258	24.5
	Every 3–6 months	165	15.7
	Annually	57	5.4
	Only when needed	571	54.3
**Healthcare visits for NCD management**	Regular check‐ups	295	28.0
	Only in emergencies	610	58.0
	Specialist consultations when needed	147	14.0
**Healthcare services utilized**	Government hospital/Clinic	433	41.2
	Private hospital/Clinic	415	39.4
	Traditional healer	30	2.9
	Pharmacy or self‐monitoring	174	16.5
**Reasons for choosing healthcare type**	Affordability	369	35.1
	Accessibility	365	34.7
	Cultural beliefs	54	5.1
	Trust in provider	264	25.1
**Barriers in accessing healthcare**	Financial constraints	501	47.6
	Distance to healthcare facility	217	20.6
	Lack of transportation	75	7.1
	Cultural/Religious beliefs	39	3.7
	Fear of stigma	51	4.8
	Lack of knowledge	169	16.1
**Reasons for delaying/Avoiding treatment**	Fear of diagnosis/Treatment	252	24.0
	Perceived low symptom severity	363	34.5
	Cost of treatment	347	33.0
	Previous negative experience	90	8.6
**Preferred healthcare provider**	General practitioner (GP)	344	32.7
	Specialist	531	50.5
	Traditional healer	127	12.1
	Others	50	4.8
**Quality of life (QoL) measures**	Overall QoL	2.99	(0.961)
	General health	2.84	(0.945)
	Physical health	52.06	(15.83)
	Psychological health	47.16	(16.76)
	Social relationships	38.83	(19.13)
	Environment	45.99	(19.03)

Abbreviation: NCD, noncommunicable disease.

### 3.3 Healthcare‐Seeking Behavior

Around 58.0% of participants visited healthcare facilities only in emergencies, whereas 28.0% reported regular check‐ups. Provider preference varied: 41.2% used government hospitals, 39.4% used private clinics, and the remainder relied on other providers, including pharmacies or informal practitioners. Affordability (35.1%) and accessibility (34.7%) were the most frequently cited reasons for provider choice, highlighting structural and financial barriers influencing HSB (Table S1).

### 3.4 Barriers to Healthcare Access

**FIGURE 2:**
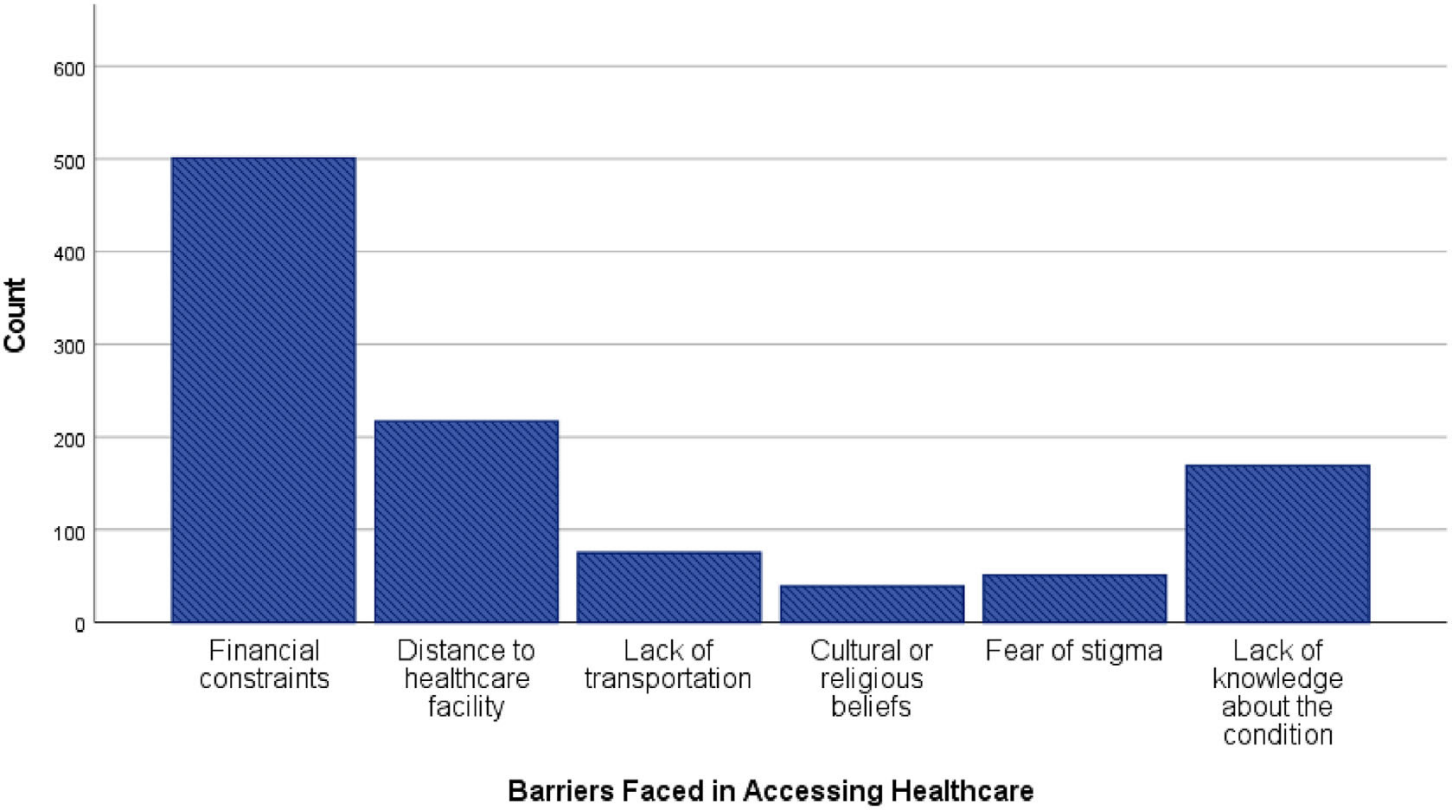
Distribution of major barriers faced in accessing healthcare among patients with NCD in Bangladesh.

Financial constraints were the most commonly reported barrier, affecting 501 participants (47.6%), followed by long distance to healthcare facilities reported by 217 participants (20.6%). Transportation difficulties were cited by 75 respondents (7.1%), whereas lack of knowledge accounted for 169 cases (16.1%). Cultural or religious beliefs (39 participants, 3.7%) and fear of stigma (51 participants, 4.8%) were less frequently reported but indicate the presence of persistent sociocultural barriers to timely healthcare utilization (Figure 2).

### 3.5 QoL Outcomes

The overall mean QoL score was 2.99 (SD = 0.96) on a 1–5 scale. The highest mean scores were observed in the physical health domain (*M* = 52.06, SD = 15.83), whereas the lowest scores were in social relationships (*M* = 38.83, SD = 19.13). Age was significantly associated with physical (*p* < 0.001) and psychological (*p* = 0.018) health, with younger participants reporting better QoL. Similarly, urban residents had significantly higher QoL scores than rural residents (*p* < 0.001), suggesting geographic disparities in access to healthcare and social support (Table S2).

### 3.6 Predictors of QoL

Socioeconomic status was positively associated with overall QoL (*β* = 0.174, *p* < 0.001) and environmental health (*β* = 0.204, *p* < 0.001), indicating the role of financial security and living conditions in shaping well‐being. Occupation negatively predicted physical health (*β* = −0.171, *p* < 0.001), suggesting work‐related stress or time constraints may reduce QoL. Gender differences were observed in psychological health (*β* = −0.109, *p* < 0.001), with males reporting better outcomes than females, potentially reflecting gendered disparities in care‐seeking and social support (Table 3).

**TABLE 3:** Regression analysis of predictors of quality of life.

Variables	Overall QoL (*β*, *p* value)	General health (*β*, *p* value)	Physical health (*β*, *p* value)	Psychological health (*β*, *p* value)	Social relationships (*β*, *p* value)	Environment (*β*, *p* value)
**Age**	0.066 (0.145)	0.120 (0.010)	0.024 (0.594)	0.132 (0.005)	0.122 (0.009)	0.192 (<0.001)
**Gender**	−0.011 (0.727)	−0.106 (0.001)	−0.043 (0.166)	−0.109 (<0.001)	−0.022 (0.495)	−0.009 (0.780)
**Educational background**	0.096 (0.011)	0.093 (0.016)	0.111 (0.003)	0.017 (0.666)	0.056 (0.144)	−0.025 (0.504)
**Socioeconomic status**	0.174 (<0.001)	0.066 (0.058)	0.042 (0.211)	0.101 (0.004)	0.076 (0.028)	0.204 (<0.001)
**Occupation**	−0.116 (0.003)	−0.161 (<0.001)	−0.171 (<0.001)	−0.125 (0.002)	−0.115 (0.004)	−0.154 (<0.001)
**Marital status**	−0.035 (0.412)	−0.061 (0.165)	−0.148 (<0.001)	−0.117 (0.008)	−0.114 (0.009)	−0.109 (0.011)
**Residential area**	−0.065 (0.040)	0.002 (0.957)	−0.071 (0.024)	−0.057 (0.079)	−0.076 (0.019)	−0.109 (<0.001)
**Primary NCD diagnosis**	0.011 (0.740)	0.029 (0.405)	−0.033 (0.326)	0.000 (0.992)	−0.031 (0.379)	−0.872 (0.383)
**Type of healthcare services utilized**	0.125 (<0.001)	0.080 (0.008)	0.093 (0.002)	0.028 (0.355)	0.105 (<0.001)	3.691 (<0.001)
**Model fit (*R* ^2^)**	0.094	0.058	0.120	0.051	0.055	0.090

Abbreviation: NCD, noncommunicable diseases.

### 3.7 Predictors of HSBs

Educational attainment consistently and positively predicted all HSB outcomes (*p* < 0.05). Occupation was inversely related to follow‐up frequency (*β* = −0.206, *p* < 0.001), healthcare visits (*β* = −0.131, *p* = 0.001), and provider preference (*β* = −0.107, *p* = 0.009), possibly reflecting employment‐related time constraints. Age significantly predicted provider preference (*β* = 0.111, *p* = 0.019), with older adults favoring specialist consultations. Marital status was inversely associated with follow‐up frequency (*β* = −0.105, *p* = 0.012). Gender, socioeconomic status, and residential area were not significant predictors in most models. Although the *R* ^2^ values were modest (0.018–0.124), the findings highlight key socioeconomic and demographic determinants influencing both HSB and QoL (Table 4). These modest *R* ^2^ values indicate that additional unmeasured contextual or health system factors may contribute to variation in HSB.

**TABLE 4:** Multivariate regression analysis of predictors of health‐seeking behavior components among patients with noncommunicable disease (NCD) in Bangladesh (*n* = 1052).

Variables	Frequency of medical follow‐ups (*β*, *p* value)	Frequency of healthcare visits for NCD management (*β*, *p* value)	Primary reasons for choosing healthcare type (*β*, *p* value)	Barriers faced in accessing healthcare (*β*, *p* value)	Reasons for delaying or avoiding treatment (*β*, *p* value)	Preferred healthcare provider for NCD management (*β*, *p* value)
**Age**	−0.002 (0.965)	−0.016 (0.739)	0.040 (0.390)	0.083 (0.077)	−0.006 (0.899)	0.111 (0.019)
**Gender**	−0.037 (0.230)	−0.022 (0.493)	−0.012 (0.713)	−0.011 (0.077)	−0.014 (0.673)	−0.025 (0.444)
**Educational background**	0.164 (<0.001)	0.171 (<0.001)	0.146 (<0.001)	0.080 (0.039)	0.142 (<0.001)	0.145 (<0.001)
**Socioeconomic status**	−0.041 (0.217)	−0.065 (0.065)	0.050 (0.155)	0.065 (0.068)	−0.034 (0.340)	−0.046 (0.189)
**Occupation**	−0.206 (<0.001)	−0.131 (0.001)	−0.069 (0.089)	−0.072 (0.080)	−0.045 (0.267)	−0.107 (0.009)
**Marital status**	−0.105 (0.012)	0.059 (0.178)	0.002 (0.959)	0.020 (0.645)	0.026 (0.556)	−0.014 (0.749)
**Residential area**	−0.012 (0.709)	0.015 (0.638)	0.049 (0.137)	−0.035 (0.288)	−0.036 (0.279)	−0.050 (0.130)
**Primary NCD diagnosis**	0.017 (0.611)	0.029 (0.417)	0.027 (0.441)	0.034 (0.339)	−0.031 (0.387)	−0.035 (0.330)
**Model fit (*R* ^2^)**	0.124	0.041	0.027	0.018	0.022	0.027

A subgroup analysis comparing younger participants (<25 years) with older adults (≥25 years) showed that education and socioeconomic status remained significant predictors of HSB in both age groups, although effect sizes were stronger among older participants. This suggests that while the sample contained a large proportion of younger respondents, the overall pattern of determinants was consistent across age strata (Table 4).

## 4 Discussion

This study examined the factors influencing HSB and QoL among patients with NCDs in Bangladesh, using Andersen's Behavioral Model as the guiding framework. To our knowledge, this is the first study in Bangladesh to jointly examine HSB and QoL under Andersen's Behavioral Model, highlighting how enabling resources such as education and occupation interact with social context. The findings reveal that a significant proportion of participants sought healthcare only in emergencies, with affordability and accessibility as primary determinants of healthcare utilization. The most common NCDs were hypertension, diabetes, and cardiovascular diseases. Socioeconomic status significantly impacted both healthcare utilization and QoL, with QoL scores highest in physical health and lowest in social relationships, underscoring the need for social support interventions. These results align with previous research on HSB and QoL among NCD patients in LMICs, particularly in Bangladesh, where financial and infrastructural barriers have been shown to hinder access to care [27]. The high proportion of respondents aged 18–24 years likely reflects the sampling locations, many of which were situated in urban and peri‐urban settings near educational institutions and employment hubs. Younger individuals in such settings may be more likely to seek diagnosis or screening, leading to their overrepresentation in facility‐based samples. Although emerging evidence suggests that NCD onset is occurring at younger ages in South Asia, this age distribution should be interpreted cautiously and may not represent the national NCD population. Limited healthcare resources in Bangladesh have historically prioritized acute infectious diseases over chronic NCDs, leaving the health system underprepared for the growing NCD burden [4]. The relatively small number of older adults limited the feasibility of robust age‐stratified regression analyses, and future studies with more balanced age distributions are warranted.

A notable finding was the overreliance on emergency care rather than preventive healthcare, consistent with Rasul et al., who reported inadequate routine check‐ups as a barrier to early diagnosis and treatment of NCDs in Bangladesh [28]. The predominance of medication‐based treatment over lifestyle modifications further highlights systemic tendencies to prioritize acute pharmacological interventions over preventive strategies, mirroring trends across LMICs [28, 29]. Low levels of awareness, education on NCD management, and prevailing cultural and societal norms further restrict proactive HSB [10].

Another important observation was the inconsistency in QoL outcomes compared to previous research. Although psychological distress is often reported as the lowest scoring QoL domain among NCD patients, our study found social relationships (mean = 38.83) to score lower than psychological health (mean = 47.16). This discrepancy may reflect the cultural context of Bangladesh, where strong family support networks offer psychological resilience despite limited broader social engagement [23]. Rural participants had significantly poorer QoL than urban participants (*p* < 0.001), consistent with global evidence showing rural populations experience greater barriers to healthcare access, leading to worse disease management and QoL outcomes [12, 30, 31]. The findings have several policy implications. Public health interventions should prioritize routine health check‐ups and screening programs to reduce reliance on emergency care. Financial barriers, reported by 47.6% of participants, highlight the need for affordable treatment options and health insurance schemes, which could lower out‐of‐pocket expenses and improve long‐term disease management. Strengthening rural healthcare infrastructure and expanding community‐based engagement programs would help address rural–urban disparities in healthcare access and outcomes. Given that medication was the prevailing treatment option, integrating lifestyle modification programs focusing on diet, physical activity, and sleep hygiene could improve long‐term prognosis. Routine counseling within healthcare services can also encourage adherence to healthy behaviors [32]. Additionally, community outreach activities such as medical camps, free health check‐ups, and screening initiatives could improve QoL, particularly in the domain of social relationships. The study also found gender disparities in psychological health, with men reporting better outcomes than women. This finding indicates the need for gender‐sensitive interventions and efforts to address social barriers limiting women's healthcare access. The observed gender difference in psychological health may reflect sociocultural dynamics prevalent in Bangladesh and similar contexts. Women often face structural barriers such as reduced financial autonomy, limited mobility without accompaniment, and disproportionate caregiving responsibilities, all of which can influence stress levels, perceived well‐being, and access to supportive healthcare resources. These structural inequalities may partially explain the lower psychological QoL scores observed among female participants.

Education emerged as a strong enabling factor consistent with Andersen's Behavioral Model, as more educated patients engaged in regular follow‐ups and proactive provider selection, likely due to better health literacy. In contrast, employment status negatively influenced HSB, possibly reflecting time constraints or competing priorities among working populations. Interestingly, although gender and residence significantly affected QoL, they were not strong predictors of HSB, demonstrating the complex nature of healthcare‐seeking behaviors and the need for tailored, context‐specific interventions. The modest *R* ^2^ values observed across regression models are consistent with behavioral health research, where complex outcomes such as healthcare utilization and QoL are influenced by numerous unmeasured contextual, cultural, and system‐level factors not captured in structured surveys.

This study has several strengths. The large sample size (*n* = 1052) allowed for robust statistical analysis and representation across diverse sociodemographic groups. The use of validated instruments such as WHOQoL‐BREF and application of Andersen's Behavioral Model enhanced conceptual rigor and comparability with international research. Inclusion of participants from both urban and rural settings across three divisions provides valuable regional insight into HSB and QoL among NCD patients in Bangladesh.

This study has several limitations. First, its cross‐sectional design precludes causal inference between predictors and outcomes. Second, reliance on self‐reported measures may introduce recall or social desirability bias, particularly for treatment adherence and lifestyle behaviors. Third, the purposive sampling strategy and high proportion of young participants limit generalizability to the broader NCD population of Bangladesh. Finally, although several socioeconomic predictors were included, other relevant determinants such as health system quality, family history, and cultural beliefs were not measured and may explain additional variance in outcomes.

## 5 Conclusion

This study highlights the substantial barriers to healthcare access and the QoL challenges faced by patients with NCDs in Bangladesh. Findings demonstrate an overreliance on emergency healthcare services, with education and employment emerging as key determinants of HSB. Financial and accessibility barriers remain critical, whereas behavioral engagement is shaped by awareness, literacy, and occupational constraints. The observed rural–urban disparities, gender‐based differences in psychological health, and preference for medication over lifestyle interventions further underscore the need for targeted and context‐specific interventions. Addressing these challenges requires strengthening healthcare policies, improving affordability through subsidized programs and health insurance, and expanding rural healthcare infrastructure. Public health efforts should prioritize preventive care through awareness campaigns, community‐based screening programs, and lifestyle counseling integrated within routine care. Future research should employ longitudinal designs to track evolving HSB patterns, evaluate the impact of health policy reforms, and explore gender disparities and social determinants of health in greater depth. Integrating lifestyle modification programs with pharmacological treatment, alongside inclusive and equitable healthcare reforms, will be essential to reducing the NCD burden and improving long‐term health outcomes in Bangladesh. By integrating behavioral models with QoL assessment, this study provides actionable evidence for designing equitable, patient‐centered NCD strategies in Bangladesh.

## Author Contributions

**Kazi Abdus Sobur:** conceptualization, investigation, visualization, formal analysis, methodology, data curation, writing – original draft, validation. **Md. Faisal Ahmed:** conceptualization and study design. All authors (Kazi Abdus Sobur, Md. Faisal Ahmed, Gazi Mahjabin Islam, Sanjana Islam, and Saiyada Hossain Saima) were involved in data curation, formal analysis, writing – original draft preparation, and writing – review and editing. All authors have read and confirmed the final draft of the manuscript.

## Funding

The authors have nothing to report.

## Ethics Statement

Formal Ethical Approval was taken from the Institutional Review Board (IRB) of the Bangladesh Institute of Innovative Health Research (protocol number‐ BIIHR‐2024‐017).

## Consent

All participants were informed about the purpose, procedures, potential risks, and benefits of the study. Informed consent was obtained from all individual participants included in the study prior to data collection.

## Conflicts of Interest

The authors declare no conflicts of interest.

## Supporting information




**Supporting file 1:** puh270238‐sup‐0001‐SuppMat1.docx

**Supporting file 2:** puh270238‐sup‐0002‐SuppMat2.docx

**Supporting file 3:** puh270238‐sup‐0003‐TableS1.docx

**Supporting file 4:** puh270238‐sup‐0004‐TableS2.docx

## Data Availability

The datasets used and/or analyzed during the current study are available from the corresponding author on reasonable request.
