# Supplementary material for: Determinants of Health‐Seeking Behavior and Quality of Life in Patients With Noncommunicable Diseases in Bangladesh
Source: Public Health Chall. 2026 Apr 21;5(2):e70238. doi: 10.1002/puh2.70238 (PMC13098756; doi:10.1002/puh2.70238)
Supplement: Supplementary file 1 — Supporting file 1: puh270238‐sup‐0001‐SuppMat1.docx [file PUH2-5-e70238-s001.docx]

**Informed Consent Form for Participation in Research Study**

**Study Title:**
*Determinants of Health-Seeking Behavior and Quality of Life in Patients with Non-Communicable Diseases in Bangladesh*

**Purpose of the Study:**
You are invited to participate in a research study that aims to explore the factors influencing how patients with non-communicable diseases (NCDs) in Bangladesh seek healthcare and how these factors affect their quality of life. Your participation will help us understand how to improve healthcare services for people like you.

**Procedures:**
If you agree to participate, you will be asked to complete a survey that includes questions about your personal background, access to healthcare, type of illness, and your quality of life. This process will take approximately **20–25 minutes**.

**Voluntary Participation:**
Your participation is completely voluntary. You have the right to refuse to answer any question or withdraw from the study at any time without any negative consequences.

**Confidentiality:**
All information you provide will be kept strictly confidential. Your name or any identifying details will not be recorded. The data will be stored securely and used only for research purposes. Results will be reported in aggregate form only.

**Risks and Benefits:**
There are no physical risks associated with participation. Some questions may be personal, but you may skip any that make you uncomfortable. While you may not benefit directly, the findings of this study may help improve healthcare programs and services for people with NCDs in the future.

**Compensation:**
You will not receive any payment for participation, but your time and contribution are greatly appreciated.

**Consent Statement:**

I have read (or been read) the information above. I have had the opportunity to ask questions, and all of my questions have been answered. I voluntarily agree to participate in this study.

- Participant’s Signature: ___________________________
- Date: _______________________
- Interviewer’s Signature (if verbal consent): ___________________________
- Date: _______________________
