# Supplementary material for: Determinants of Health‐Seeking Behavior and Quality of Life in Patients With Noncommunicable Diseases in Bangladesh
Source: Public Health Chall. 2026 Apr 21;5(2):e70238. doi: 10.1002/puh2.70238 (PMC13098756; doi:10.1002/puh2.70238)
Supplement: Supplementary file 2 — Supporting file 2: puh270238‐sup‐0002‐SuppMat2.docx [file PUH2-5-e70238-s004.docx]

**STROBE Checklist for Cross-Sectional Studies**

**Study Title:** Factors Influencing Health-Seeking Behavior and Quality of Life in Patients with Non-Communicable Diseases in Bangladesh

**Title and Abstract**

1. **(a)** Study design mentioned in title and abstract as "cross-sectional"
2. **(b)** Abstract includes a clear summary of objectives, methods, findings, and conclusions

**Introduction**
2. **Background/Rationale:** Clearly explained in the Introduction section (para 1-2) with global and Bangladeshi NCD context
3. **Objectives:** Explicit objective stated at the end of the Introduction and Abstract; based on Andersen's Behavioral Model

**Methods**
4. **Study Design:** Cross-sectional design noted at the beginning of the Methods section (2.2)
5. **Setting:** Urban and rural healthcare facilities in Dhaka, Khulna, and Mymensingh divisions, between Nov 2024–Feb 2025
6. **Participants:** Inclusion/exclusion criteria, sampling method (stratified purposive), and total sample size (n=1052) provided in section 2.3
7. **Variables:** Defined across four subsections—sociodemographic, NCD-specific data, health-seeking behavior, and QoL (2.4)
8. **Data sources/measurement:** Explained in 2.4 and 2.5, including validated tools (Bangla WHOQoL-BREF) and interviewer-administered questionnaire
9. **Bias:** Interviewer training and standardized procedures to reduce interviewer and recall bias noted in 2.5
10. **Study size:** Sample size calculation using Cochran's formula described in 2.3
11. **Quantitative variables:** Handling described in 2.6 with dummy coding for categorical variables and regression models
12. **Statistical methods:**

- **(a)** Described in 2.6: Chi-square, t-tests, ANOVA, regression models
- **(b)** Subgroup comparisons for gender, SES, urban/rural noted in results tables and text
- **(c)** Missing data strategy not specifically mentioned (could be clarified)
- **(d)** Sampling strategy acknowledged in 2.3 (purposive, stratified)
- **(e)** Sensitivity analyses not conducted

**Results**
13. **Participants:** Total participants (n=1052) reported in 3.1, with inclusion flow implied through eligibility criteria; flow diagram not included
14. **Descriptive data:** Detailed in Tables 1 and 2; subgroup characteristics included in Table 3 and others
15. **Outcome data:** Provided through Tables 4–6 and Figure 2 (barriers)
16. **Main results:**

- **(a)** Unadjusted and adjusted results given in regression tables
- **(b)** Categories for continuous variables (e.g., age groups) clearly reported
- **(c)** No conversion to absolute risk reported (not relevant for this study type)

1. **Other analyses:** Subgroup analyses by gender, SES, residential area; no sensitivity analysis done

**Discussion**
18. **Key results:** Summarized at the beginning of the Discussion section
19. **Limitations:** Addressed toward the end of the Discussion, including bias, generalizability, and cross-sectional limits
20. **Interpretation:** Balanced, contextualized interpretation of findings
21. **Generalisability:** External validity is discussed, especially regarding rural-urban and socioeconomic disparities

**Other Information**
22. **Funding:** Declared as "no funding received" in Funding Statement

*This checklist has been completed following the STROBE Statement (www.strobe-statement.org).*
