# Supplementary material for: Determinants of Health‐Seeking Behavior and Quality of Life in Patients With Noncommunicable Diseases in Bangladesh
Source: Public Health Chall. 2026 Apr 21;5(2):e70238. doi: 10.1002/puh2.70238 (PMC13098756; doi:10.1002/puh2.70238)
Supplement: Supplementary file 3 — Supporting file 3: puh270238‐sup‐0003‐TableS1.docx [file PUH2-5-e70238-s002.docx]

**Supplementary Table 1: Healthcare-Seeking Behaviors of Study Participants**

| **Variables** | **Gender (n, %)** | | | **Socioeconomic Status (n, %)** | | | | **Residential Area (n, %)** | | |
| --- | --- | --- | --- | --- | --- | --- | --- | --- | --- | --- |
|  | **Male** | **Female** | $\boldsymbol{\chi}$**^2^ (p)** | **Lower Class** | **Middle Class** | **Higher Class** | $\boldsymbol{\chi}$**^2^ (p)** | **Urban** | **Rural** | $\boldsymbol{\chi}$**^2^ (p)** |
| ***Primary NCD Diagnosis*** | | | | | | | | | | |
| Diabetes | 120 (47.2) | 134 (52.8) | 161.011 (<0.001) | 48 (18.9) | 173 (68.1) | 33 (13.0) | 84.650 (<0.001) | 178 (70.1) | 76 (29.9) | 20.362 (0.001) |
| Hypertension | 208 (54.6) | 173 (45.4) |  | 28 (7.3) | 324 (85.0) | 29 (7.6) |  | 304 (79.8) | 77 (20.2) |  |
| Cardiovascular Disease | 151 (81.6) | 34 (18.4) |  | 26 (14.1) | 151 (81.6) | 8 (4.3) |  | 141 (76.2) | 44 (23.8) |  |
| Cancer | 8 (47.1) | 9 (52.9) |  | 8 (47.1) | 5 (29.4) | 4 (23.5) |  | 9 (52.9) | 8 (47.1) |  |
| Chronic Respiratory Disease | 25 (47.2) | 28 (52.8) |  | 3 (5.7) | 48 (86.8) | 4 (7.5) |  | 40 (75.5) | 13 (24.5) |  |
| Others | 23 (14.2) | 139 (85.8) |  | 6 (3.7) | 151 (93.2) | 5 (3.09) |  | 138 (85.2) | 24 (14.8) |  |
| ***Duration of Illness (in years)*** | | | | | | | | | | |
| 0 to 2 | 276 (52.0) | 255 (48.0) | .640 (0.887) | 46 (8.7) | 461 (86.8) | 24 (4.5) | 51.826 (<0.001) | 414 (78.0) | 117 (22.0) | 1.122 (0.772) |
| 3 to 5 | 163 (49.2) | 168 (50.8) |  | 49 (14.8) | 253 (76.4) | 29 (8.8) |  | 255 (77.0) | 76 (23.0) |  |
| 6 to 8 | 47 (50.0) | 47 (50.0) |  | 7 (7.4) | 78 (83.0) | 9 (9.6) |  | 70 (74.5) | 24 (25.5) |  |
| 9 to more | 49 (51.0) | 47 (49.0) |  | 17 (17.7) | 58 (60.4) | 21 (21.9) |  | 71 (74.0) | 25 (26.0) |  |
| ***Current Treatment Type*** | | | | | | | | | | |
| Medication | 272 (45.9) | 321 (54.1) | 15.266 (0.002) | 61 (10.3) | 481 (81.1) | 51 (8.6) | 18.944 (0.004) | 436 (73.5) | 157 (26.5) | 10.958 (0.012) |
| Lifestyle Modification | 172 (56.4) | 133 (43.6) |  | 42 (13.8) | 241 (79.0) | 22 (7.2) |  | 254 (83.3) | 51 (16.7) |  |
| Surgery | 17 (50.0) | 17 (50.0) |  | 6 (17.6) | 21 (61.8) | 7 (20.6) |  | 26 (76.5) | 8 (23.5) |  |
| Others | 74 (61.7) | 46 (38.3) |  | 10 (8.3) | 107 (89.2) | 3 (2.5) |  | 94 (78.3) | 26 (21.7) |  |
| ***Frequency of Medical Follow-ups*** | | | | | | | | | | |
| Monthly | 125 (48.4) | 133 (51.6) | 3.779 (0.286) | 32 (12.4) | 200 (77.5) | 26 (10.1) | 6.661 (0.353) | 190 (73.6) | 68 (26.4) | 3.531 (0.317) |
| Every 3-6 Months | 78 (47.3) | 87 (52.7) |  | 18 (10.9) | 132 (80.0) | 15 (9.1) |  | 131 (79.4) | 34 (20.6) |  |
| Annually | 26 (45.6) | 31 (54.4) |  | 10 (17.5) | 43 (75.4) | 4 (7.0) |  | 41 (71.9) | 16 (28.1) |  |
| Only When Needed | 306 (53.6) | 265 (46.4) |  | 59 (10.3) | 474 (83.0) | 38 (6.7) |  | 447 (78.3) | 124 (21.7) |  |
| ***Frequency of Healthcare Visits for NCD Management*** | | | | | | | | | | |
| Regular Check-ups | 149 (50.5) | 146 (49.5) | .879 (0.644) | 35 (11.9) | 225 (76.3) | 35 (11.9) | 10.950 (0.027) | 233 (79.0) | 62 (21.0) | 7.724 (0.021) |
| Only in emergencies | 306 (50.2) | 304 (49.8) |  | 71 (11.6) | 503 (82.5) | 36 (5.9) |  | 453 (74.3) | 157 (25.7) |  |
| Specialist Consultations When Needed | 80 (54.4) | 67 (45.6) |  | 13 (8.8) | 122 (83.0) | 12 (8.2) |  | 124 (84.4) | 23 (15.6) |  |
| ***Types of Healthcare Services Utilized*** | | | | | | | | | | |
| Government Hospital/Clinic | 224 (51.7) | 209 (48.3) | 4.070 (0.254) | 74 (17.1) | 345 (79.7) | 14 (3.2) | 65.785 (<0.001) | 314 (72.5) | 119 (27.5) | 21.036 (<0.001) |
| Private Hospital/Clinic | 200 (48.2) | 215 (51.8) |  | 15 (3.6) | 349 (84.1) | 51 (12.3) |  | 349 (84.1) | 66 (15.9) |  |
| Traditional Healer | 13 (43.3) | 17 (56.7) |  | 7 (23.3) | 23 (76.7) | 0 (0.0) |  | 19 (63.3) | 11 (36.7) |  |
| Pharmacy or Self-monitoring | 98 (56.3) | 76 (43.7) |  | 23 (13.2) | 133 (76.4) | 18 (10.3) |  | 128 (73.6) | 46 (26.4) |  |
| ***Primary Reasons for Choosing Healthcare Type*** | | | | | | | | | | |
| Affordability | 199 (53.9) | 170 46.1) | 4.381 (0.223) | 65 (17.6) | 290 (78.6) | 14 (3.8) | 56.231 (<0.001) | 271 (73.4) | 98 (26.6) | 16.108 (0.001) |
| Accessibility | 172 (47.1) | 193 (52.9) |  | 41 (11.2) | 301 (82.5) | 23 (6.3) |  | 269 (73.7) | 96 (26.3) |  |
| Cultural Beliefs | 31 (57.4) | 23 (42.6) |  | 5 (9.3) | 41 (75.9) | 8 (14.8) |  | 46 (85.2) | 8 (14.8) |  |
| Trust in Provider | 133 (50.4) | 131 (49.6) |  | 8 (3.0) | 218 (82.6) | 38 (14.4) |  | 224 (84.8) | 40 (15.2) |  |
| ***Barriers Faced in Accessing Healthcare*** | | | | | | | | | | |
| Financial Constraints | 257 (51.3) | 244 (48.7) | 4.916 (0.426) | 72 (14.4) | 396 (79.0) | 33 (6.6) | 29.649 (<0.001) | 369 (73.7) | 132 (26.3) | 7.604 (0.179) |
| Distance to Healthcare Facility | 107 (49.3) | 110 (50.7) |  | 13 (6.0) | 186 (85.7) | 18 (8.3) |  | 174 (80.2) | 43 (19.8) |  |
| Lack of Transportation | 36 (48.0) | 39 (52.0) |  | 12 (16.0) | 59 (78.7) | 4 (5.3) |  | 56 (74.7) | 19 (25.3) |  |
| Cultural or Religious Beliefs | 22 (56.4) | 17 (43.6) |  | 7 (17.9) | 32 (82.1) | 0 (0.0) |  | 32 (82.1) | 7 (17.9) |  |
| Fear of Stigma | 20 (39.2) | 31 (60.8) |  | 4 (7.8) | 39 (76.5) | 8 (15.7) |  | 41 (80.4) | 10 (19.6) |  |
| Lack of Knowledge about the Condition | 93 (55.0) | 76 (45.0) |  | 11 (6.5) | 138 (81.7) | 20 (11.8) |  | 138 (81.7) | 31 (18.3) |  |
| ***Reasons for Delaying or Avoiding Treatment*** | | | | | | | | | | |
| Fear of Diagnosis or Treatment | 121 (48.0) | 131 (52.0) | 2.906 (0.406) | 42 (16.7) | 189 (75.0) | 21 (8.3) | 26.460 (<0.001) | 177 (70.2) | 75 (29.8) | 12.317 (0.006) |
| Perceived Severity of Symptoms is Low | 181 (49.9) | 182 (50.1) |  | 20 (5.5) | 321 (88.4) | 22 (6.1) |  | 293 (80.7) | 70 (19.3) |  |
| Cost of Treatment | 189 (54.5) | 158 (45.5) |  | 48 (13.8) | 266 (76.7) | 33 (9.5) |  | 264 (76.1) | 83 (23.9) |  |
| Previous Negative Experience with Healthcare | 44 (48.9) | 46 (51.1) |  | 9 (10.0) | 74 (82.2) | 7 (7.8) |  | 76 (84.4) | 14 (15.6) |  |
| ***Preferred Healthcare Provider for NCD Management*** | | | | | | | | | | |
| General Practitioner (GP) | 166 (48.3) | 178 (51.7) | 2.016 (0.569) | 48 (14.0) | 284 (82.6) | 12 (3.5) | 51.867 (<0.001) | 237 (68.9) | 107 (31.1) | 34.345 (<0.001) |
| Specialist | 278 (52.4) | 253 (47.6) |  | 33 (6.2) | 440 (82.9) | 58 (10.9) |  | 444 (83.6) | 87 (16.4) |  |
| Traditional Healer | 63 (49.6) | 64 (50.4) |  | 31 (24.4) | 86 (67.7) | 10 (7.9) |  | 86 (67.7) | 41 (32.3) |  |
| Others | 28 (56.0) | 22 (44.0) |  | 7 (14.0) | 40 (80.0) | 3 (6.0) |  | 810 (77.0) | 242 (23.0) |  |
