# Supplementary material for: Determinants of Health‐Seeking Behavior and Quality of Life in Patients With Noncommunicable Diseases in Bangladesh
Source: Public Health Chall. 2026 Apr 21;5(2):e70238. doi: 10.1002/puh2.70238 (PMC13098756; doi:10.1002/puh2.70238)
Supplement: Supplementary file 4 — Supporting file 4: puh270238‐sup‐0004‐TableS2.docx [file PUH2-5-e70238-s003.docx]

**Supplementary Table 2: Quality of Life Outcomes Across Different Domains**

| **Variables** | **Overall QoL** | | **General Health** | | **Physical Health** | | **Psychological Health** | | **Social Relationship** | | **Environment** | |
| --- | --- | --- | --- | --- | --- | --- | --- | --- | --- | --- | --- | --- |
|  | **Mean (SD)** | **t/F (p-value)** | **Mean (SD)** | **t/F (p-value)** | **Mean (SD)** | **t/F (p-value)** | **Mean (SD)** | **t/F (p-value)** | **Mean (SD)** | **t/F (p-value)** | **Mean (SD)** | **t/F (p-value)** |
| ***Age (in years)*** | | | | | | | | | | | | |
| 18 to 24 | 3.12 (0.05) | 4.226 (<0.001) | 2.95 (0.05) | 2.936 (0.012) | 55.65 (0.77) | 11.026 (<0.001) | 48.77 (0.83) | 2.739 (0.018) | 40.66 (0.86) | 3.132 (0.008) | 46.78 (0.88) | 3.552 (0.003) |
| 25 to 34 | 2.89 (0.06) |  | 2.75 (0.06) |  | 51.68 (1.01) |  | 45.20 (1.06) |  | 36.34 (1.17) |  | 42.59 (1.25) |  |
| 35 to 44 | 2.81 (0.09) |  | 2.67 (0.08) |  | 48.02 (1.25) |  | 44.19 (1.49) |  | 36.67 (1.64) |  | 45.12 (1.79) |  |
| 45 to 54 | 3.09 (0.09) |  | 2.92 (0.09) |  | 49.07 (1.31) |  | 49.32 (1.55) |  | 41.30 (1.72) |  | 50.17 (1.97) |  |
| 55 to 64 | 3.03 (0.11) |  | 2.77 (0.11) |  | 47.80 (1.56) |  | 47.63 (1.63) |  | 40.21 (1.89) |  | 50.53 (1.86) |  |
| 65 to older | 2.70 (0.13) |  | 2.74 (0.09) |  | 44.31 (1.81) |  | 46.30 (1.80) |  | 35.31 (2.22) |  | 45.54 (1.99) |  |
| ***Gender*** | | | | | | | | | | | | |
| Male | 3.02 (0.04) | 0.934 (0.35) | 2.94 (.04) | 3.605 (<0.001) | 53.22 (0.70) | 2.417 (0.016) | 49.13 (0.73) | 3.908 (<0.001) | 39.68 (0.77) | 1.534 (0.125) | 46.43 (0.80) | 0.750 (0.454) |
| Female | 2.97 (0.04) |  | 2.74 (.04) |  | 50.86 (0.67) |  | 45.12 (0.72) |  | 37.96 (0.81) |  | 45.45 (0.86) |  |
| ***Educational Background*** | | | | | | | | | | | | |
| No Formal Education | 2.46 (.13) | 15.074 (<0.001) | 2.47 (0.10) | 6.986 (<0.001) | 46.17 (1.34) | 14.725 (<0.001) | 43.93 (1.89) | 3.621 (0.006) | 34.38 (1.89) | 6.577 (<0.001) | 45.36 (2.17) | 4.860 (<0.001) |
| Primary | 3.00 (.12) |  | 2.91 (0.11) |  | 44.91 (1.88) |  | 47.07 (1.78) |  | 35.6 (2.50) |  | 44.41 (2.49) |  |
| Secondary | 2.67 (.12) |  | 2.51 (0.11) |  | 46.17 (1.71) |  | 43.72 (1.96) |  | 37.97 (2.32) |  | 42.16 (2.43) |  |
| Higher Secondary | 2.83 (.06) |  | 2.79 (0.06) |  | 49.69 (0.95) |  | 45.29 (1.06) |  | 35.41 (1.14) |  | 42.61 (1.27) |  |
| Graduate and above | 3.17 (.04) |  | 2.94 (0.04) |  | 55.04 (0.66) |  | 48.76 (0.69) |  | 41.22 (0.73) |  | 48.13 (0.74) |  |
| ***Socioeconomic Status*** | | | | | | | | | | | | |
| Lower Class | 2.53 (0.08) | 26.463 (<.001) | 2.62 (0.08) | 4.097 (0.017) | 47.03 (1.05) | 6.963 (<.0001) | 44.15 (1.41) | 6.225 (0.002) | 36.08 (1.47) | 7.093 (<0.001) | 41.94 (1.55) | 31.885 (<0.001) |
| Middle Class | 3.01 (0.03) |  | 2.86 (0.03) |  | 52.78 (0.56) |  | 47.05 (0.58) |  | 38.57 (0.64) |  | 45.08 (0.65) |  |
| Higher Class | 3.49 (0.09) |  | 2.96 (0.09) |  | 51.94 (1.52) |  | 52.51 (1.67) |  | 45.46 (1.62) |  | 61.18 (1.68) |  |
| ***Occupation*** | | | | | | | | | | | | |
| Unemployed | 2.70 (0.15) | 9.278 (<0.001) | 2.83 (0.13) | 7.759 (<0.001) | 51.61 (1.87) | 22.186 (<0.001) | 46.81 (2.19) | 4.621 (0.001) | 38.00 (2.23) | 3.853 (0.004) | 44.74 (2.37) | 3.639 (0.006) |
| Student | 3.17 (0.04) |  | 3.01 (0.04) |  | 56.70 (0.75) |  | 49.36 (0.80) |  | 40.95 (0.82) |  | 47.51 (0.85) |  |
| Homemaker | 2.80 (0.07) |  | 2.64 (0.06) |  | 47.26 (0.94) |  | 44.86 (1.13) |  | 38.20 (1.38) |  | 46.53 (1.46) |  |
| Employed | 2.86 (0.06) |  | 2.70 (0.06) |  | 49.05 (0.91) |  | 44.77 (0.98) |  | 35.74 (1.09) |  | 42.9 (1.16) |  |
| Retired | 3.13 (0.12) |  | 2.83 (0.09) |  | 43.40 (2.01) |  | 49.18 (1.93) |  | 40.00 (2.22) |  | 50.34 (2.28) |  |
| ***Marital Status*** | | | | | | | | | | | | |
| Single | 3.13 (0.04) | 9.775 (<0.001) | 2.99 (0.04) | 10.478 (<0.001) | 56.50 (0.69) | 32.151 (<0.001) | 49.32 (0.76) | 6.690 (<0.001) | 41.17 (0.76) | 7.292 (<0.001) | 47.63 (0.80) | 4.327 (0.005) |
| Married | 2.82 (0.04) |  | 2.68 (0.04) |  | 47.34 (0.64) |  | 44.92 (0.70) |  | 36.42 (0.83) |  | 44.06 (0.88) |  |
| Divorced | 3.00 (0.28) |  | 2.53 (0.30) |  | 47.90 (3.28) |  | 41.18 (4.48) |  | 30.20 (4.21) |  | 40.81 (4.71) |  |
| Widowed | 3.40 (0.24) |  | 3.13 (0.29) |  | 45.95 (5.03) |  | 46.39 (5.27) |  | 40.89 (5.28) |  | 53.96 (4.75) |  |
| ***Residential Area*** | | | | | | | | | | | | |
| Urban | 3.07 (0.03) | 4.491 (<0.001) | 2.86 (0.03) | 1.208 (0.227) | 53.12 (0.57) | 4.005 (<0.001) | 47.92 (0.61) | 2.701 (0.007) | 39.88 (0.64) | 3.458 (<0.001) | 47.42 (0.69) | 4.487 (<0.001) |
| Rural | 2.75 (0.06) |  | 2.78 (0.06) |  | 48.51 (0.88) |  | 44.61 (0.94) |  | 35.32 (1.14) |  | 41.22 (1.06) |  |
| ***Primary NCD Diagnosis*** | | | | | | | | | | | | |
| Diabetes | 2.99 (0.06) | 2.805 (0.016) | 2.82 (0.054) | 1.485 (0.192) | 48.93 (0.83) | 6.147 (<0.001) | 47.88 (0.94) | 2.138 (0.059) | 39.37 (1.07) | 1.167 (0.323) | 48.73 (1.14) | 2.857 (0.014) |
| Hypertension | 2.94 (0.05) |  | 2.77 (0.05) |  | 52.58 (0.85) |  | 45.69 (0.90) |  | 38.18 (0.94) |  | 43.72 (0.98) |  |
| Cardiovascular Disease | 3.02 (0.07) |  | 2.97 (0.07) |  | 54.59 (1.17) |  | 49.50 (1.17) |  | 39.60 (1.35) |  | 47.35 (1.34) |  |
| Cancer | 2.71 (0.22) |  | 2.76 (0.20) |  | 44.75 (3.68) |  | 42.40 (4.35) |  | 35.29 (4.34) |  | 46.14 (4.14) |  |
| Chronic Respiratory Disease | 2.75 (0.13) |  | 2.81 (0.13) |  | 47.24 (2.04) |  | 44.26 (2.31) |  | 34.59 (2.77) |  | 42.04 (2.95) |  |
| Others | 3.20 (0.08) |  | 2.92 (0.08) |  | 55.20 (1.34) |  | 48.23 (1.41) |  | 40.41 (1.43) |  | 46.76 (1.54) |  |
| ***Type of Healthcare Services Utilized*** | | | | | | | | | | | | |
| Government Hospital/Clinic | 2.74 (0.05) | 23.183 (<0.001) | 2.72 (0.05) | 7.197 (<0.001) | 49.65 (0.73) | 7.646 (<0.001) | 45.27 (0.81) | 4.125 (.006) | 35.41 (0.92) | 9.695 (<0.001) | 41.53 (0.94) | 15.077 (<0.001) |
| Private Hospital/Clinic | 3.20 (0.04) |  | 2.91 (0.05) |  | 53.22 (0.81) |  | 49.15 (0.82) |  | 41.27 (0.85) |  | 49.83 (0.92) |  |
| Traditional Healer | 2.53 (0.16) |  | 2.50 (0.13) |  | 49.40 (2.86) |  | 44.44 (2.68) |  | 36.67 (3.82) |  | 44.17 (3.35) |  |
| Pharmacy or Self-monitoring | 3.20 (0.06) |  | 3.04 (0.07) |  | 55.75 (1.14) |  | 47.58 (1.24) |  | 41.92 (1.20) |  | 48.26 (1.24) |  |
